# Structural insights into tetraspanin CD9 function

**DOI:** 10.1038/s41467-020-15459-7

**Published:** 2020-03-30

**Authors:** Rie Umeda, Yuhkoh Satouh, Mizuki Takemoto, Yoshiko Nakada-Nakura, Kehong Liu, Takeshi Yokoyama, Mikako Shirouzu, So Iwata, Norimichi Nomura, Ken Sato, Masahito Ikawa, Tomohiro Nishizawa, Osamu Nureki

**Affiliations:** 1 Department of Biological Sciences Graduate School of Science, The University of Tokyo, 7-3-1 Hongo, Bunkyo-ku, Tokyo Japan; 2 Laboratory of Molecular Traffic, Institute for Molecular and Cellular Regulation (IMCR), Gunma University, Maebashi, 371-8512 Japan; 3 Research Institute for Microbial Diseases, Osaka University, 3-1 Yamadaoka, Suita, Osaka Japan; 4 Department of Cell Biology, Graduate School of Medicine, Kyoto University, Kyoto, Japan; 5 Laboratory for Protein Functional and Structural Biology, RIKEN Center for Biosystems Dynamics Research, 1-7-22 Suehiro-cho, Tsurumi-ku, Yokohama, Kanagawa 230-0045 Japan; 6 RIKEN SPring-8 Center, 1-1-1 Kouto, Sayo-cho, Sayo-gun, Hyogo Japan; 7 Gunma University Initiative for Advanced Research (GIAR), Gunma University, Maebashi, 371-8512 Japan; 8 Precursory Research for Embryonic Science and Technology (PRESTO), Japan Science and Technology, Bunkyo-ku, Tokyo Japan; 9 Present Address: Preferred Networks, Inc., Bunkyo-ku, Tokyo Japan

**Keywords:** Cell biology, Electron microscopy, X-ray crystallography

## Abstract

Tetraspanins play critical roles in various physiological processes, ranging from cell adhesion to virus infection. The members of the tetraspanin family have four membrane-spanning domains and short and large extracellular loops, and associate with a broad range of other functional proteins to exert cellular functions. Here we report the crystal structure of CD9 and the cryo-electron microscopic structure of CD9 in complex with its single membrane-spanning partner protein, EWI-2. The reversed cone-like molecular shape of CD9 generates membrane curvature in the crystalline lipid layers, which explains the CD9 localization in regions with high membrane curvature and its implications in membrane remodeling. The molecular interaction between CD9 and EWI-2 is mainly mediated through the small residues in the transmembrane region and protein/lipid interactions, whereas the fertilization assay revealed the critical involvement of the LEL region in the sperm-egg fusion, indicating the different dependency of each binding domain for other partner proteins.

## Abstract (web summary)

Tetraspanins play critical roles in various physiological processes, ranging from cell adhesion to virus infection. Here authors report the crystal structure of CD9 and the cryo-electron microscopic structure of CD9 in complex with its single membrane-spanning partner protein, EWI-2.

## Introduction

The tetraspanin protein family members are ubiquitously expressed in higher organisms and involved in a wide range of physiological processes, such as cell motility and adhesion, tumor invasion, fertilization, and virus infection^1–4^. Despite their broad involvement in physiological processes, the knock-out of the tetraspanin gene results in only moderate phenotypes, indicating their functional redundancy, except for some tissue-specific subtypes, such as peripherins in the retina^5^ and uroplakins in the bladder^6^. The importance of tetraspanins was highlighted by the infertile phenotype of the *Cd9* KO female mice^7–9^, but its molecular function; specifically how CD9 is involved in sperm-egg binding/fusion, has remained poorly understood. Based on biochemical studies, tetraspanins are proposed to form complex protein networks in biological membranes, by recruiting other partner proteins into the tetraspanin-enriched microdomain (TEM). Therefore, tetraspanin is considered as a molecular organizer that associates with the partner proteins to exert their cellular functions. However, a recent study has challenged the classical view of the tetraspanin network, showing that each tetraspanin cluster consists of only a small number of molecules and only partially overlaps with the localization of its binding partner, MHC-II, in B-cells^10^. Therefore, further studies will be required to elucidate their physiological functions.

The tetraspanin proteins share the same membrane topology of four membrane-spanning domains and the first and second extracellular loops, termed the short extracellular loop (SEL) and large extracellular loop (LEL), respectively (Supplementary Fig. 1). The recently reported crystal structure of a tetraspanin protein, CD81, revealed a reversed teepee-like arrangement of the four transmembrane (TM) helices, which create a central pocket in the intramembranous region^11^. The molecular dynamics (MD) simulation, together with the mutation analysis, suggested that cholesterol binding at the central cavity modulates CD81 association with its partner protein, CD19^11^. However, the means by which tetraspanins form complex protein networks in cell membranes remain poorly understood. While the LEL is implicated in molecular associations with partner proteins, the detailed interactions are still unclear, and thus the associations of tetraspanins with broad members of single membrane-spanning proteins, including integrins, immunoglobulin superfamily proteins, and TGF-β receptors, and how tetraspanins regulate their functions are not well understood. Here, we report the crystal structure of CD9 and the cryo-electron microscopy (cryo-EM) structure of CD9 in complex with its partner protein, EWI-2. Combined with the mutational analysis of CD9 in mouse egg fertilization, we show that the broad interactions through TM3 and the LEL are important for the molecular associations of tetraspanins, which are critical for their functions.

## Results

### Crystal structure of human CD9

We first crystallized wild-type human CD9 by the lipidic cubic phase (LCP) method, but the obtained crystals diffracted to about only 20 Å. Considering the possibility that the molecular flexibility of CD9 hinders the tight crystal packing interactions, we made several series of truncated constructs, and the construct that lacked part of the LEL region (Thr175-Lys179) and the C-terminal tail (Glu226-Val228) yielded good quality crystals, which diffracted to over 3.0 Å^12^. For the experimental phase determination, we introduced an additional cysteine residue at Ile20 and co-crystallized the protein with methyl-mercury (I) chloride. The structure was determined by the single isomorphous replacement with anomalous scattering (SIRAS) method and refined to 2.7 Å resolution (Supplementary Table 1).

**Fig. 1:**
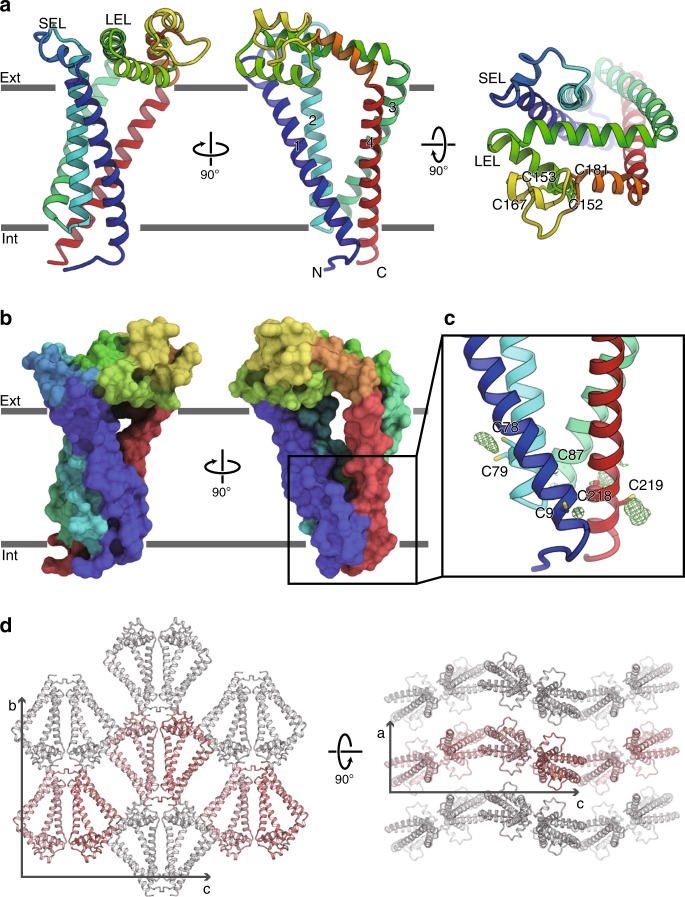
Crystal structure of human CD9. **a** Overall structure of human CD9, viewed from the membrane plane (left and middle) and from the extracellular side (right). The transmembrane helices and the two extracellular loops (SEL and LEL) are labeled. Cys152-Cys181 and Cys153-Cys167 form disulfide bonds. **b** Surface representation of CD9, colored according to **a**. **c** Palmitoylation of the cytoplasmic cysteine residues. Green meshes show *F*_o_−*F*_c_ densities contoured at 2.5 σ, indicating the palmitoylation of the cysteine residues on the cytoplasmic end of the four transmembrane helices. **d** Crystal packing of CD9, showing the curvature generation by the asymmetric shape of the CD9 molecules. Pairs of CD9 protomers are aligned up and down alternatively along the *c*-axis, resulting in the wavy lipid layers of the LCP crystal.

Human CD9 is folded into four-transmembrane helices, with their intracellular ends tightly bundled together and their extracellular ends loosely packed with each other, creating a large central cavity inside the intramembranous region (Fig. 1a–c). The short extracellular loop (SEL) between TM1 and TM2 and the large extracellular loop (LEL) between TM3 and TM4 extend over this central cavity. The LEL is stabilized by disulfide bond pairs between the highly conserved cysteine residues (Cys152-Cys181 and Cys153-Cys167) (Fig. 1a). We observed extra densities near the cysteine residues on the intracellular ends of the TM helices (Cys9, Cys79, Cys87, and Cys219), which are probably derived from the attached palmitoyl moieties, consistent with the heterogeneous bands of the purified CD9 protein observed in the SDS-PAGE analysis^12^. These lipid modifications probably anchor the TM helices on the lipid membrane and stabilize the TM bundle at the intracellular end. CD9 shares high sequence similarity (approximately 60%) with the related tetraspanin protein CD81, and accordingly, the overall structure of CD9 is essentially similar to the previously reported structure of CD81^11^ (Supplementary Fig. 2). Nonetheless, the current high-resolution CD9 structure allowed the modeling of the entire molecule, including the SEL, which were not modeled in the previous CD81 structure due to the high anisotropy of the crystal. While the extracellular loops (SEL and LEL) have diverged sequences, the transmembrane region is highly conserved among the tetraspanin family members (Supplementary Fig. 1). Therefore, this arrangement of the four transmembrane helices in the CD9 and CD81 structures might represent the common architecture of the tetraspanin family proteins.

The current LCP crystal of CD9 revealed a unique feature: due to the cone-like molecular shape, the arrayed molecules in the lipidic environment generate curvature in the lipid membranes, resulting in wavy layers in the crystalline lattice (Fig. 1d). Several studies have shown the localization of tetraspanin proteins in specific regions of curved cell membranes. For instance, CD9 and its related tetraspanin, CD81, are localized in the growing tips of virus buds and involved in the infection cycle, and are directly implicated in generating membrane curvature^13,14^. Molecular shapes of membrane proteins are highly associated with the protein localization as well as membrane shaping and remodeling^15–18^. Therefore, the asymmetric shape of CD9 and the consequent curvature in the LCP crystal may explain its accumulation into specific regions with a low curvature radius. Furthermore, several studies have suggested clustering of tetraspanins in biological membranes^19^, and such clustering could generate local membrane curvature and thus induce reorganization of cell membranes, as previously indicated^20^. Although our hypothesis will require future elucidation, this is in good agreement with the previous study showing that the knock-out of the *Cd9* gene results in the abnormal distribution and shapes of microvilli in oocytes^21^.

### MD simulation

**Fig. 2:**
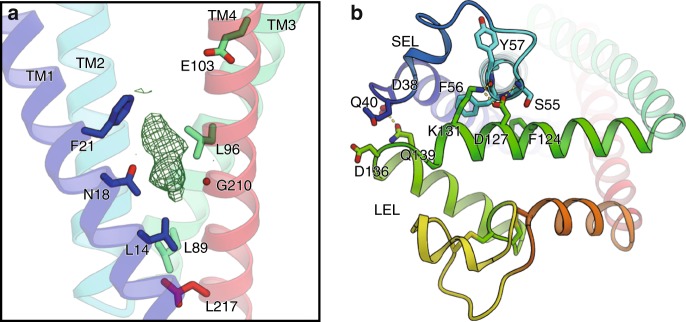
Central hydrophilic pocket and extracellular loops. **a** The central cavity is rendered hydrophilic by the conserved residues, Asn18 and Glu103. The critical residue for cholesterol binding, Glu219 in CD81, is replaced by Gly210 in CD9 (Supplementary Fig. 1). The *F*_o_−*F*_c_ map contoured at 2.5 sigma shows an unknown density within the cavity, probably derived from the monoolein present during the lipidic cubic phase crystallization. **b** The central cavity is sealed on the extracellular side by the association between the SEL and LEL. The residues involved in the interactions are shown, and the hydrogen bonding interactions are indicated by yellow dotted lines.

**Fig. 3:**
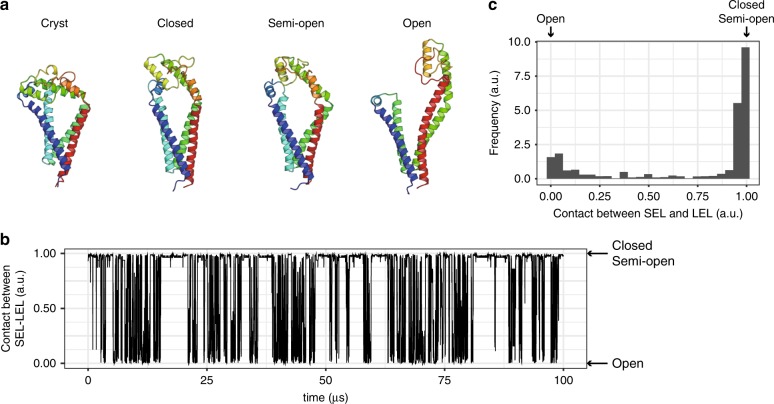
MD simulation of CD9. **a** Structural comparison between the crystal structure (Cryst) and the three major conformations in the MD simulation (Closed, Semi-open, and Open). **b** Time course of the contacts between the SEL and LEL in the reconstructed 100 μs trajectory. The value of 1 corresponds to the shorter distance between the SEL and LEL, and the value of 0 corresponds to the longer distance between the SEL and LEL. For the detailed definition of the contacts between the SEL and LEL, see Methods section. **c** Distribution of the contacts between the SEL and LEL in the longer reconstructed trajectory, corresponding to the 15 ms time scale.

Within the central cavity, hydrophilic residues, such as Asn18 and Glu103, are located in the middle of the transmembrane helices and generate a hydrophilic intramembranous environment (Fig. 2a), and the cavity is sealed on the extracellular side by the association between the two extracellular loops, SEL and LEL (Fig. 2b). To investigate the molecular behavior of CD9, we performed a MD simulation combined with a Markov State Model (MSM) analysis, which yielded a reconstructed trajectory corresponding to about a 20 μs time scale (Fig. 3a–c and Supplementary Fig. 3, Supplementary Movies 1, 2). The simulation showed the conformational flexibilities of the SEL and LEL, resulting in frequent transitions between the major states, referred to as the closed, semi-open, and open conformations (Fig. 3a). In the closed conformation, the LEL weakly associates with the SEL and remains beside the membrane plane, as in the crystal structure, while in the open conformation, the LEL adopts an up position, apart from the SEL. A similar conformation change was also reported in the CD81 simulation, but CD81 adopted the closed conformation only when cholesterol is bound at the central cavity, and quickly shifted to the open conformation without cholesterol in the simulation. In contrast, in the current CD9 simulation, we observed spontaneous transitions between the open and closed conformations even in the absence of cholesterol. Consistently, the critical amino acid for the cholesterol binding (Glu219 in CD81) is not conserved in the other tetraspanin members and is replaced with Gly210 in CD9 (Fig. 2a), while the residues lining the central cavity are highly conserved among the tetraspanin family members (Supplementary Fig. 1). Nevertheless, we observed an electron density within the cavity in the current closed CD9 structure, even though cholesterol was not added during the crystallization (Fig. 2a). It is highly possible that the monoolein molecules used in the LCP crystallization or endogenous lipids are accommodated in the cavity, thus suggesting the regulatory roles of lipid molecules in the CD9 function^22^.

### Complex structure of CD9 with EWI-2

**Fig. 4:**
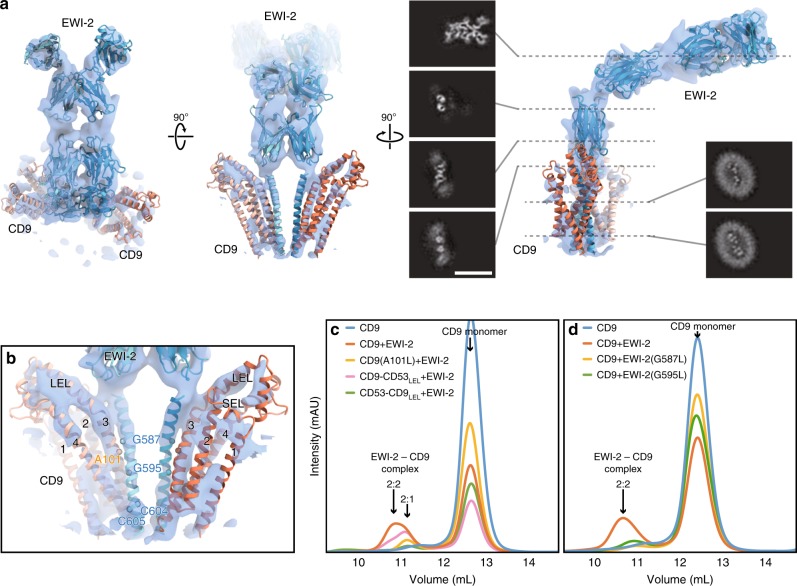
Cryo-EM structure of CD9 in complex with EWI-2. **a** Cryo-EM density map of the Fab-CD9-EWI-2 complex. The density for the Fab fragment was somewhat disordered (Supplementary Fig. 6). Homology models of the Ig-like domains of EWI-2 were constructed with the PHYRE2 server^80^ and roughly fitted into the density. The TM helices and the two extracellular loops (SEL and LEL) of the CD9 crystal structure are separately fitted into the density. The density for the detergent micelle is omitted for clarity. Black panels show sections of the density, demonstrating the C2 symmetry in the TM region and the proximal Ig-like domain. Scale bar, 100 Å. **b** Close-up view of the TM region. CD9 adopts a semi-open conformation with a slight rearrangement of the LEL, as compared to the crystal structure (Supplementary Fig. 6). The residues in the EWI-2 membrane-spanning region were modeled according to the mutant analyses in Fig. 2c, d. **c**, **d** Complex formation assays for CD9 mutants (**c**) and EWI-2 mutants (**d**). GFP-labeled CD9 and EWI-2 were co-expressed in HEK cells, and complex formation was analyzed by FSEC. The slight peak shift toward the smaller molecular weight indicates the complex formation with 2:1 stoichiometry.

Tetraspanin proteins are considered to form complicated interaction networks in cells to exert their functions, by associating and organizing other functional proteins within these networks. To understand this mechanism, we performed a structural analysis of CD9 in complex with its major partner protein, EWI-2. EWI family proteins belong to a immunoglobulin superfamily (IgSF), containing a single membrane pass with multiple (4–8) Ig-like domains, and associate with broad members of the tetraspanin proteins, including CD9, CD81, and CD151^23,24^. Although their precise functions have not been clarified, they reportedly link tetraspanins to the cytoskeleton and play regulatory roles in TGF-β and integrin signaling via the tetraspanin network^25–28^. Consistent with the previous reports^24,29^, CD9 forms a tight complex with EWI-2, when the proteins are expressed together in HEK cells (Supplementary Fig. 4a). The complex was purified in digitonin micelles and subjected to a single particle cryo-EM analysis, and the structure was resolved at 8.2 Å resolution with the Fourier shell correlation (FSC) gold standard criteria (Supplementary Fig. 5a, b; Supplementary Table 2). The cryo-EM map clearly revealed the pair of the tandem Ig-like domains of the two EWI-2 protomers extending in parallel from the detergent micelle, indicating the dimeric architecture of EWI-2. Although extra densities probably corresponding to the CD9 extracellular loops were observed, the precise location of the CD9 molecules could not be determined in the blurred density derived from the detergent micelle (Supplementary Fig. 5a). To further improve the alignments in the single particle analysis, an anti-CD9 Fab fragment was attached to the complex (Supplementary Fig. 4b), and the cryo-EM map of the Fab-CD9-EWI-2 complex was resolved at a slightly improved resolution (7.3 Å) (Fig. 4a and Supplementary Fig. 5c, d), which successfully visualized the separate densities for the TM helices of EWI-2 and CD9, whereas the densities for the Fab fragments are substantially disordered in the final refined map (Supplementary Fig. 5c). In the complex structure, two protomers of EWI-2 form a tight dimer at the center, accompanied by two CD9 protomers sandwiching the EWI-2 dimer, with a 2:2 hetero-tetrameric stoichiometry.

Although the low resolution of the cryo-EM map hampered the atomic modeling of the overall CD9-EWI-2 complex, the four Ig-like domains and the membrane-spanning region of EWI-2 could be roughly fitted into the density map. The arrangement of the four transmembrane helices of CD9 is in good agreement with that in the LCP crystal structure, whereas the LEL adopts a slightly different conformation similar to that observed in the semi-open conformation in the MD simulation (Supplementary Fig. 6). The membrane-spanning helix of EWI-2 is located proximal to the TM3–4 helices of CD9, and is likely involved in the complex interaction (Fig. 4b). To validate the complex interactions, we made several CD9 mutants and tested their complex formation with EWI-2 by the fluorescence-detection size-exclusion chromatography (FSEC) assay (Fig. 4c). We first tested the chimeric constructs with a related tetraspanin, CD53, which belongs to the same branch of the tetraspanin family (Supplementary Fig. 1) but does not form a complex with EWI-2. The exchange of the LEL region with CD53 (CD9-CD53_LEL_) only moderately affected the complex formation, whereas the similar exchange of the LEL region of CD53 with CD9 (CD53-CD9_LEL_) failed to form the complex (Fig. 4c), indicating that the interaction between CD9 and EWI-2 is essentially mediated through the transmembrane region. Based on the amino acid sequence comparison between CD9 and CD53 (Supplementary Fig. 1), the single mutation of A101L, located on the outer side of TM3, abolished the complex formation (Fig. 4c). We further investigated the complex interaction by introducing a mutation in the membrane-spanning region of EWI-2, which revealed that the G587L and G595L mutations, within a Gly-zipper motif, show decreased complex formation (Fig. 4d). As these residues are located in the middle of the membrane-spanning region (Supplementary Fig. 7), the complementary arrangement of the small residues at the interface between CD9 and the EWI-2 is an important factor for the complex formation. In addition, a previous study showed the critical implication of the palmitoylation at the two consecutive cysteine residues of EWI-2, located on the cytoplasmic end of the membrane-spanning region, in the molecular association with CD81^30^ (Fig. 4b and Supplementary Fig. 7).Therefore, the lipid anchoring and/or protein–lipid interactions might also contribute to the interactions between tetraspanins and their binding partners. It should also be noted that, while the LEL-swapping mutant (CD9-CD53_LEL_) can still form the complex with EWI-2, this mutant shows a slight peak shift toward a smaller molecular weight in the fluorescent size-exclusion chromatography (FSEC) analysis, probably derived from the different stoichiometry of the complex formation (Fig. 4c; 2:1 of EWI-2 and CD9), suggesting its weaker interaction with EWI-2. In the current cryo-EM map, the LEL likely adopts an open conformation, approaching the juxta-membrane Ig-like domain of EWI-2 (Supplementary Fig. 6). Therefore, the TM region and the LEL are both involved in the complex interaction.

### In vitro fertilization assay

**Fig. 5:**
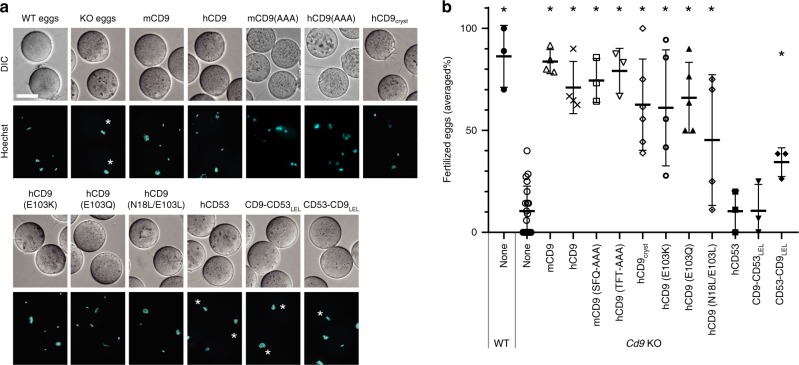
Sperm–egg fusion complementation assay. **a** Functional complementation of *Cd9* KO mouse eggs by mRNA injection. The mRNA, encoding the WT or mutants of mouse CD9, human CD9, or chimeric constructs between human CD9 and CD53, was injected into the mouse *Cd9* KO eggs. The fusion of mouse spermatozoa was visualized by the transfer of DNA dye from the eggs to the sperm nuclei. Asterisks indicate unfertilized eggs. Scale bar, 50 µm. **b** Fertilization rates in the sperm–egg fusion assay. The averaged fertilization rates of repetitive experiments are shown with error bar of standard deviation. Each dot represents one experiment. Asterisks indicate significant differences from the *Cd9* KO eggs (two-tailed, unpaired Student’s *t*-test, *P < 0.005). The number of experiments and eggs are indicated in Supplementary Fig. 8c and also provided as a Source Data file.

Tetraspanin can associate with a wide range of proteins, enabling functional variance in different cell types, and tetraspanin shows different levels of molecular association according to the binding partners^19,31^. We next investigated the molecular role of CD9 in the fertilization process, which is the most important function of CD9, although the precise roles of CD9 and its binding partners in fertilization have remained unidentified so far^32,33^. Consistent with the previous studies, the eggs derived from *Cd9* KO mice lacked fertilizing ability, which could be reverted by injections of mRNAs encoding wild-type human and mouse CD9 (Fig. 5a, b and Supplementary Fig. 8). The crystallization construct CD9_cryst_ also restored the sperm-fusing ability of the *Cd9* KO eggs, indicating that the partial truncation of the LEL region and the C-terminal tail does not affect its function in fertilization. We next investigated the mutants of the conserved hydrophilic residues within the central pocket (Fig. 2b), but the in vitro fertilization assay revealed the less important roles of these residues in the fertilization, as the N18L/E103L, E103K, and E103Q mutants only moderately affected the CD9 function in fertilization (Fig. 5b). For further investigation, we tested the fertilization competencies of chimeric constructs between CD9 and the fertilization incompetent tetraspanin member CD53, which were used in the complex formation assay with EWI-2. Swapping of the LEL region (CD9-CD53_LEL_) diminished the competency, while the swapping of the LEL region of CD53 with CD9 (CD53-CD9_LEL_) partly retained the competency (Fig. 5b), indicating the critical role of the LEL in the sperm-egg fusion by CD9, in contrast to the complex formation assay with EWI-2 (Fig. 4c). A previous study reported that alanine replacement of specific SFQ residues (173–175) in the flexible region of the LEL severely defected the fertilization competency of mouse CD9^34^, whereas this sequence is not conserved in human CD9 (Thr175, Phe176, and Thr177). To further examine the importance of the this region, we constructed the mouse and human CD9 mutants, in which the corresponding residues are replaced with alanine (SFQ and TFT to AAA, respectively), and tested their fertilization competencies (Fig. 5b). Surprisingly, both mutants (mCD9(AAA) and hCD9(AAA)) could rescue the fertilization ability of the Cd9 KO oocytes, comparable to wild type mouse and human CD9, contrary to the previous report. Consistently, the crystallized construct that completely lacks the corresponding region (Thr175–Lys179) similarly rescued the fertilization ability. Overall, our result shows dispensable role of the flexible region but an important role of the other region of the LEL for fertilization^34^. Discrepancy between the present study and the previous study about the AAA replacement is presumably due to the difference in fertilization efficiency and/or protein expression level, since in the previous study, even injections of the wild type mCd9 or hCD9 mRNAs rescued only about half of the fertilization efficiency^34^. It is still noteworthy that the fertilization competency of CD53-CD9_LEL_ is also decreased by about half as compared to the wild-type CD9, suggesting that the SEL and TM domains also contribute to the CD9 function in fertilization. These results will revise conventional view of the tetraspanin function that the flexible loop region serves as the docking site for the partner proteins, thus providing important insights into the tetraspanin function. The partner proteins of CD9 in fertilization have not yet been identified, and EWI-2 in eggs is dispensable^32,33^. However, the current fertilization assay revealed the critical involvement of the LEL region, as well as the partial involvement of other regions, showing a different hot spot of CD9 for the unidentified partner proteins, as compared to the association with EWI-2.

## Discussion

**Fig. 6:**
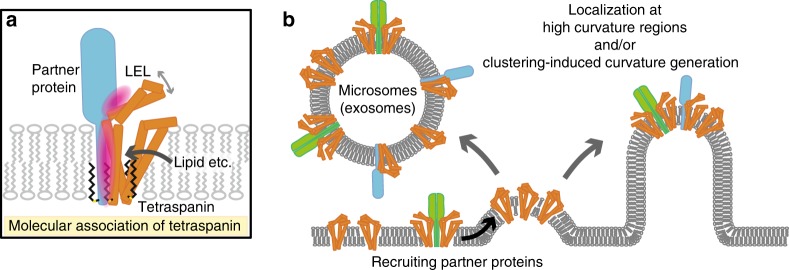
Hypothetical model of the tetraspanin function. **a** The molecular association of tetraspanin and its partner proteins is mediated through both the TM and LEL regions, but its dependency is different among the partner proteins. Lipid binding to the central cavity could modulate the molecular association by affecting the LEL conformation. **b** The highly asymmetric shape of the tetraspanins suggests their localization at high curvature regions and/or clustering-induced curvature generation. Partner proteins should also be recruited and sorted to the respective regions, such as the microvilli tips and microsomes (exosomes).

The tetraspanin proteins reportedly interact with broad members of single-spanning membrane proteins, including integrin, a disintegrin and metalloproteinases (ADAMs), TGF-β receptors, and major histocompatibility complex II (MHC-II)^4,19^, with different levels of molecular association^19,31^. The current cryo-EM structure of the CD9-EWI-2 complex and the mutation analysis revealed the importance of the small residues in the transmembrane region for the complex formation between CD9 and EWI-2. Small residues, such as Gly and Ala, are often present in the membrane-spanning regions of single membrane-spanning proteins, and are implicated in protein dynamics, helix-helix packing interactions, and thus in homo- and hetero-dimerization^35–38^. Therefore, the molecular association observed in the CD9-EWI-2 complex might be conserved in the complex interactions with other binding partners, which may explain the broad specificity for the partner proteins and the regulatory roles of tetraspanins in their partner protein functions. The cryo-EM structure and the mutation analysis revealed the partial involvement of the LEL in the complex formation with EWI-2. The current study shows the different dependencies of the LEL and TM regions for the EWI-2 association and fertilization, implying the presence of different hot spots for their partner proteins. This multi-platform binding of CD9 could explain the different levels of the molecular association by tetraspanins, although we cannot exclude the possibility that CD9 has a completely different binding site for the unidentified partner protein in fertilization. The CD9 structures and the MD simulation further suggest other factors affecting the molecular interactions, such as the association between the SEL and LEL and binding of lipid molecules to the central cavity, similar to the previous report on CD81, in which cholesterol binding to the central cavity affected the molecular interaction with its binding partner, CD19^11^ (Fig. 6a).

CD9 is abundantly expressed in mouse eggs and localized in the microvilli tips, where they are involved in microvilli formation and sperm–egg fusion^21^. Tremendous studies have suggested network formation of tetraspanin proteins in cellular membranes, but this view is still controversial, and a recent study showed colocalization of only a few tetraspanin molecules^10^. The current study suggests a tetraspanin function that the molecular shape of tetraspanin is directly involved in membrane curvature generation and specific localization at high curvature regions (Fig. 6b), as proposed for other membrane proteins^16^. Notably, this asymmetric shape is also preserved in the complex of CD9 and EWI-2, indicating that CD9 can recruit other single TM-spanning proteins into the same regions by forming heterocomplexes. In the small vesicular exosomes, tetraspanins are the most abundant proteins and play important roles in exosome production, protein biogenesis, and vesicular cargo sorting, thus determining the exosome function^39^. The current study suggests two possible roles of tetraspanin in exosome function. Firstly, the clustering of tetraspanin molecules itself may facilitate exosome budding by inducing membrane curvature, and secondly, the association with tetraspanins may direct other functional proteins into the vesicles and thus control vesicular cargo sorting, as previously proposed^13,39^ (Fig. 6b). It should also be noted that, in the current complex structure, the Ig-like domains of EWI-2 are parallel to the membrane plane, and bent perpendicular to the TM helix, thereby allowing additional molecular associations at the juxtamembrane region. This suggests the possible involvements of the EWI family proteins in indirect tetraspanin associations, by bridging between tetraspanins and other proteins, and thus in the vesicular cargo selection or the formation of complex protein networks, consistent with the previous studies^40,41^. Overall, the current structures of CD9 and its complex with EWI-2 will provide a framework to understand the molecular associations and functions of tetraspanins.

## Methods

### Crystallization of CD9

Cloning and protein purification were performed as in the previous report^12^. The human CD9 gene (UniProt accession code: P21926) was cloned into a modified pFastBac1 expression vector (Thermo Fischer Scientific), which includes an N-terminal His_8_-tag, a GFP-tag, and a tobacco etch virus (TEV) protease cleavage site. For the crystallization, the five extracellular-loop residues and the three C-terminal residues of CD9 were deleted by a PCR-based method. For mercury derivatization, a cysteine mutation was introduced by site-directed mutagenesis.

For protein expression, these modified CD9 constructs were expressed in Sf9 cells (Thermo Fischer Scientific), using the Bac-to-Bac baculovirus expression system. Sf9 cells cultured in Sf900II medium (Thermo Fischer Scientific) were infected at a density of approximately 3 × 10^6^ cells ml^−1^ and incubated for 48 h at 27 °C. The cells were collected by centrifugation (5000×*g*, 12 min), and the following procedures were performed at 4 °C or on ice. The cells were resuspended in 50 mM HEPES, pH 7.0, 150 mM NaCl, and protease inhibitors (1.7 µg ml^−1^ aprotinin, 0.6 µg ml^−1^ leupeptin, 0.5 µg ml^−1^ pepstatin, and 1 mM phenylmethysulfonyl fluoride). The cells were disrupted by sonication, and the cell debris was removed by centrifugation (4000×*g*, 10 min). The supernatant was ultra-centrifuged (186,000×*g*, 1 h), and the membrane fraction was collected and resuspended in 50 mM HEPES, pH 7.0, containing 150 mM NaCl.

The membrane fraction was solubilized in 10 mM HEPES, pH 7.0, 150 mM NaCl, 1.5% (w/v) *n*-dodecyl-β-d-maltoside (DDM), and 0.3% (w/v) cholesteryl hemisuccinate (CHS) for 1 h, and the solubilized proteins were purified by the following three chromatography steps. The insoluble material was removed by ultracentrifugation (186,000×*g*, 30 min). The supernatant was mixed with TALON Metal Affinity Resin (Clontech), and incubated for 30 min. After the incubation, the resin was washed with seven column volumes of 10 mM HEPES, pH 7.0, 150 mM NaCl, 0.1% DDM, 0.02% CHS, and 20 mM imidazole. The protein sample was then eluted with three column volumes of 10 mM HEPES, pH 7.0, 150 mM NaCl, 0.1% DDM, 0.02% CHS, and 300 mM imidazole. The eluted sample was mixed with His-tagged TEV protease (purified in-house) to cleave the His_8_-GFP tag, and dialyzed against 10 mM HEPES, pH 7.0, 150 mM NaCl, 0.1% DDM, and 0.02% CHS to remove the imidazole. After overnight dialysis, the sample was mixed with 5 ml of Ni-NTA Superflow resin (QIAGEN), and incubated for 10 min at 4 °C, to remove the His_8_-GFP tag and TEV protease. The collected flow-through fraction was then concentrated using an Amicon Ultra filter (molecular mass cut-off 30 kDa, Millipore), and further purified by gel filtration (Superdex 200 Increase 10/300 GL, GE Healthcare) in 10 mM HEPES, pH 7.0, 150 mM NaCl, 0.05% DDM, and 0.01% CHS. The peak fractions were concentrated to approximately 15 mg ml^−1^, using an Amicon Ultra filter (molecular mass cut-off 50 kDa, Millipore). The SDS-PAGE analysis shows a higher molecular weight aggregation that is formed during denaturing by SDS^12^. The truncated and cysteine mutants were purified by the same procedure as described above.

The purified CD9 samples were reconstituted into the lipidic cubic phase (LCP) by mixing with liquefied monoolein (Sigma) in a 2:3 protein to lipid ratio (w/v), using the twin-syringe mixing method^42^. For the sandwich-drop crystallization, aliquots of the protein-LCP mixture (50 nl) were dispensed onto 96-well glass plates and overlaid with the precipitant solution (800 nL), using a Gryphon LCP robot (Art Robbins Instruments, LLC). The best crystals were obtained in a reservoir solution consisting of 32–38% PEG 200 and 10–50 mM MOPS, pH 6.5, or a similar solution containing 10–50 mM Tris-HCl, pH 7.5, instead of MOPS. For the preparation of the mercury-derivative crystals, the co-crystallization of CD9 with CH_3_HgCl was performed. CH_3_HgCl was dissolved in DMSO, and added to the protein samples at a final concentration of 2 mM. After an incubation for 20 min on ice, the samples were crystallized by the LCP method as described above. The best crystals were obtained in a reservoir solution similar to that used for the native crystals. The crystals were collected within 3 weeks, using MicroMounts or mesh grid loops (MiTeGen), and flash cooled in liquid nitrogen.

### Molecular dynamics simulation

The truncated region of the LEL (Thr175-Lys179) was modeled using Modeller^43^, using the CD81 structure as the template. All cysteine residues on the TM helices (Cys9, Cys79, Cys87, and Cys219) were palmitoylated, as in the crystal structure. All of the water molecules observed in the crystal structure were kept. The missing hydrogen atoms were built with the program VMD^44^. The protein was embedded into the phosphoryloleoylphosphatidylcholine (POPC) lipid bilayer, using the MemProtMD pipeline^45^. The net charge of the simulation system was neutralized through the addition of 150 mM NaCl. The simulation system was 104 × 105 × 107 Å and contained 118,038 atoms. The molecular topologies and parameters from the Charmm36 force field^46,47^ were used for the protein, lipid, and water molecules. For the palmitoylated Cys residue, the molecular topology and parameters were obtained from the NAMD wiki (https://www.ks.uiuc.edu/Research/namd/wiki/index.cgi?ParameterTopologyRepository), and converted to the Gromacs format with the charmm2gromacs-pvm.py script (http://www.gromacs.org/@api/deki/files/185/=charmm2gromacs-pvm.py). Molecular dynamics simulations were performed with the program Gromacs 5.1.4^48^. First, energy minimization was performed using the steepest descent, with a cut-off of 1000 kJ mol^–1^ nm^–1^ with the position restraints to the non-hydrogen atoms in the protein with a force constant of 1000 kJ mol^–1^. Next, random velocities were assigned according to a Maxwell distribution at a temperature of 310 K for each atom, and an equilibration run (eq1) was performed for 100 ps in the canonical (NVT) ensemble (310 K, 104 × 105 × 107 Å volume) with the same restraints as in the minimization step. Next, an equilibration run (eq2) was performed for 6 ns in the isothermal–isobaric (NPT) ensemble (310 K, 1 bar) with the same restraints. Finally, the system was equilibrated in the NPT ensemble (310 K, 1 bar) for 5 ns, with the position restraints for the C-alpha atoms of the protein. Production runs (prod1) were performed in the NPT ensemble (310 K, 1 bar) without any position restraints. We repeated the procedure from eq1 to prod1 50 times, with different initial velocities at eq1. The simulation time of prod1 ranged from 100 to 1000 ns, and the total sum of the simulation time was 8.42 μs.

To widely sample the conformational space, we performed clustering analyses for these trajectories and started the MD simulations from the selected cluster centers. Each conformation in the trajectories was featurized by the atomic positions of its C-alpha atoms (superimposed onto the crystal structure). The dimensions of these features were reduced to 15 with principal component analysis (PCA), followed by k-means clustering into 2000 clusters. Subsequently, 100 clusters were randomly selected, and the structure nearest to the cluster center was selected as the initial state of the MD simulation. The 100 ns long MD simulation was performed for each structure, following the random initial velocity assignment and 1 ns equilibration in the NPT ensemble. Finally, we obtained aggregated 18.42 μs trajectories, subsampled at 0.1 ns for a total of about 180,000 molecular structures.

We performed the MSM analyses for these trajectories. Various featurizations for the protein conformation, which describe the dynamics of the system, were assessed, including the atomic positions of the backbone atoms, the dihedral angles of the protein backbone, and so on. The appropriate featurization method was selected by the 3-fold cross validation method with variational GMRQ objective functions^49^, and finally we selected an RMSD based featurization: the RMSD values for the 50 reference structures were used as the feature. The 50 reference structures were selected from the cluster center of the k-medoid clustering over all obtained trajectories. The RMSD values were converted by the following function: ${\mathrm{exp}}[ - {\mathrm{RMSD}}^2/2{\upsigma}^2]$ with sigma = 0.5. Next, the trajectories were discretized into 500 clusters by performing the k-means clustering based on the 50-dimensional features, followed by building the MSM with the lagtime = 20 ns (see Supplementary Fig. 3a for the implied timescale plots of the MSM). The hyperparameters, such as the number of clusters in the k-means clustering, were also determined by maximizing the GMRQ score, except for the lagtime of the MSM calculation. The contact between SEL and LEL (Fig. 3b, c) is defined as follows: ${\mathrm{Contact}} = \left( {1 - \left( {d{\mathrm{/}}d_0} \right)^n} \right)/\left( {1 - \left( {d/d_0} \right)} \right.^m$ where *d* is the minimum distance between ECL1 and ECL2, *d*_0_ is the cutoff distance, and *n* and *m* are some integers. In this paper, *d*_0_ = 8.0 Å, *n* = 6, and *m* = 12 was used. For the 2-dimensional visualization in Supplementary Fig. 3, the principal component analysis (PCA) was performed for the 50-dimensional feature space.

Constant temperature was maintained by using V-rescaling^50^ with a time constant of 0.1 ps in eq1, and a Nosé-Hoover thermostat^51,52^ with a time constant of 0.5 ps in eq2 and the production runs. Pressure was controlled with semi-isotropic coupling to a Parrinello–Rahman barostat^53^, with a time constant of 5.0 ps and a compressibility of 4.5 × 10^−5^ bar^–1^. The LINCS algorithm^54^ was used for bond constraints. Long range electrostatic interactions were calculated with the particle mesh Ewald method^55^. The simulation results were analyzed and visualized with mdtraj^56^, MSMbuilder3^57^, MSMExplorer1.1.0^58^, scikit-learn^59^, and ggplot2^60^.

### Data collection and structure determination and refinement

All diffraction datasets were collected using the micro-focused X-ray beam at SPring-8 BL32XU^61^. The microcrystals in the loop were identified by a raster scan and analysis by SHIKA^62^. Small wedge data, each consisting of 10–180°, were collected from single crystals. The collected datasets were processed automatically with KAMO^63^. Each dataset was indexed and integrated using XDS^64^, followed by a hierarchical clustering analysis using the correlation coefficients of the normalized structure amplitudes between data sets. Finally, a group of outlier-rejected data sets were scaled and merged using XSCALE^64^.

The structure was determined by the single isomorphous replacement with anomalous scattering (SIRAS) method, using the program AutoSHARP^65^. The model construction and refinement were performed using the programs COOT and PHENIX^66^. The data collection and refinement statistics are summarized in Supplementary Table 1. All molecular graphics were prepared using CueMol (http://www.cuemol.org/).

### In vitro fertilization

The cDNA encoding human CD9, tagged at the N terminus with enhanced green fluorescent protein (EGFP), was cloned between the *Xba*I and *Xho*I sites of the pCAGGS vector^67^. The mutations were introduced by a PCR-based method. T7 promoter-tagged cDNAs were amplified by PCR with the primers (5′-GGGTAATACGACTCACTATAGGGGCAACGTGCTGGTTGTTGTGC-3′ and 5′-AGCCAGAAGTCAGATGCTCAAGGGGCTTC-3′) and high fidelity Taq polymerase, using the pCAGGS vectors as templates. RNA synthesis, poly-A tail addition, and further preparation for egg injection were performed as previously described, using a mMESSAGE mMACHINE T7 kit (Ambion) and a Poly(A) Tailing kit (Ambion) according to the manufacturer’s instructions.

Gene complementation experiments using *Cd9* deficient eggs were performed basically according to the previous study^68^. Immature, germinal vesicle eggs were collected from the ovaries of wild-type B6D2F1 or *Cd9* KO female mice (10–20 weeks old), 46 h after the injection of anti-inhibin antibody solution (CARD HyperOva, Kyudo). Antral follicles were suspended in FHM medium supplemented with 250 μM dibutyryl-cyclic AMP (dbcAMP), and punctured with 26 G needles (Sigma). After dissociating the cumulus cells by pipetting, the eggs were transferred to the medium supplemented with 250 μM dbcAMP and 5 μg ml^−1^ cytochalasin B (Sigma), and then the injections of mRNAs (100 ng µl^−1^) into the eggs were performed using a piezo-micromanipulator with a glass capillary needle. The eggs were washed three times, and then cultured in TYH medium containing 10% fetal bovine serum (Biowest) for in vitro maturation. After 14 h, the zona were removed from the MII eggs, using a micromanipulator^69^. Pre-loading of Hoechst 33342 into the denuded eggs was performed, as previously described^70^, and after three washes with fresh TYH medium, the eggs were subjected to confocal image acquisition with the low-invasive imaging system^71^, and only the eggs with adequate EGFP fluorescence intensities on the oolemma were used for the fusion assay. In one experiment, considering the failure of superovulation, 2–4 *Cd9* KO female mice were assigned to an experimental group and 2 WT mice were assigned to a control group.

Hoechst 33342 pre-loaded eggs were inseminated with 2 × 10^5^ cells ml^−1^ B6D2F1 capacitated sperm, for 30 min. After a quick wash in kSOM medium, the eggs were subjected to confocal imaging. Confocal images were acquired as described^68^. Experiments were repeated at least three times for each batch. Total numbers of inseminated eggs per batch (28–150) are indicated in Supplementary Fig. 8c.

### Protein expression and purification for cryo-EM

The human wild type CD9 gene was cloned into the pEG BacMam vector^72^, with an N-terminal His_8_-tag, a GFP-tag, and a tobacco etch virus (TEV) protease cleavage site. The human EWI-2 (UniProt accession: Q969P0) wild type genes were cloned from human brain complementary DNA (ZYAGEN) into the pEG BacMam vector, with a C-terminal Flag epitope (DYKDDDDK)-tag.

CD9 and EWI-2 were co-expressed in HEK293S GnTI- (N-acetylglucosaminyl-transferase I-negative) cells (American Type Culture Collection, catalog no. CRL-3022), using the BacMam expression system^72^. For protein expression, HEK293S GnTI- cells cultured in FreeStyle medium (Thermo Fischer Scientific) were infected at a density of approximately 3 × 10^6^ cells ml^−1^ and incubated at 37 °C. After 16 h, sodium butyrate was added to the cells, which were further incubated for 48 h at 30 °C. The membrane fractions were collected by the same procedure as described above, and solubilized in 10 mM HEPES, pH 7.0, 150 mM NaCl, and 1% digitonin (Calbiochem) for 1 h at 4 °C. Insoluble materials were removed by ultracentrifugation (186,000×*g*, 30 min). The supernatant was mixed with ANTI-FLAG M2 Affinity gel (Sigma), and incubated for 1 h. After the incubation, the resin was washed with ten column volumes of 10 mM HEPES, pH 7.0, 150 mM NaCl, and 0.1% digitonin. The protein sample was then eluted with three column volumes of 10 mM HEPES, pH 7.0, 150 mM NaCl, 0.1% digitonin, and Flag peptide (0.1 mg ml^−1^). The eluted sample was mixed with CNBr-Activated Sepharose 4 Fast Flow beads (GE Healthcare) coupled with an anti-GFP nanobody (GFP enhancer)^73^, and incubated for 1 h at 4 °C. The beads were washed with ten column volumes of 10 mM HEPES, pH 7.0, 150 mM NaCl, and 0.1% digitonin, and further incubated overnight with TEV protease to cleave the His_8_-GFP tag. The collected flow-through fraction was then concentrated with an Amicon Ultra filter (molecular mass cut-off 50 kDa, Millipore), and further purified by gel filtration (Superose 6 Increase 10/300 GL column, GE Healthcare) in 10 mM HEPES, pH 7.0, 150 mM NaCl, and 0.1% digitonin.

The mouse-derived IgG antibody Fab fragment that specifically binds to human CD9 was prepared according to the previous method^74,75^. To prepare the complex with Fab fragments, the concentrated sample was mixed with the Fab fragments at a 1:0.4 weight ratio and incubated on ice for 1 h before the gel filtration. The peak fractions of these proteins were collected and concentrated to 5 mg ml^−1^. A 3 µl portion of the concentrated sample was applied to a glow-discharged Quantifoil R1.2/1.3 Cu/Rh 300 mesh grid (Quantifoil), blotted using a Vitrobot Mark IV (FEI) under 6 °C and 100% humidity conditions, and then frozen in liquid ethane.

### EM image acquisition and data processing

The grid images of the CD9-EWI-2 and Fab-CD9-EWI-2 complexes were obtained with a Talos Arctica transmission electron microscope (FEI) operated at 200 kV, and recorded by a K2 Summit direct electron detector (Gatan) with a physical pixel size of 1.352 Å. The dataset was acquired with the EPU software (FEI). Each image was dose fractionated to 40 frames at a dose rate of 6–8 e^−^ pixel^−1^ per second, to accumulate a total dose of ~50 e^−^ Å^−2^. In total, 2360 and 1398 movies were collected for the Fab-CD9-EWI-2 CD9-EWI-2 complexes, respectively. The movie frames were aligned in 5 × 5 patches and dose weighted in MotionCor2^76^. Defocus parameters were estimated by CTFFIND 4.1^77^. For the analysis of the Fab-attached complex, a total of 542,954 particles were picked up by Laplacian-of-Gaussian based auto-picking implemented in REILON 3.0 was performed, and extracted in 4.23 Å pixel^−1^. These particles were subjected to two-dimensional classification with the Ignore CTFs until first peak option, and selected classes were further classified in 3D in RELION 3.0^78,79^. Subsequently, 124,701 good particles were selected, re-extracted in original pixel size (1.352 Å pixel^−1^), and subjected to Bayesian polishing implemented in RELION 3.0 and 3D auto-refine with the mask excluding the Fab densities. Postprocessing yielded a 7.3 Å resolution map, based on the gold-standard (FSC = 0.143) criteria. The analysis of the CD9-EWI-2 complex was performed in a similar manner but without Bayesian polishing, because it did not improve the density. Briefly, a total of 276,879 particles was initially extracted and 82,071 particles were used for final reconstruction, with the pixel size of 2.82 Å pixel^−1^. The overall gold-standard resolution was 8.2 Å.

### FSEC assay

The interaction between CD9 and EWI-2 was analyzed by fluorescence-detection size-exclusion chromatography (FSEC). HEK293T adhesion cells (0.4 × 10^6^ ml^−1^) were cultured in 6-well plates (2000 µl per well) with DMEM containing 10% FBS for 1 day, and transfected with 2 µg of the CD9-encoding pEG expression plasmid with a GFP-tag and 2 µg of the EWI-2-encoding pEG expression plasmid with a FLAG-tag, using 5 µl of Lipofectamine 2000 transfection reagent (Thermo Fisher), according to the manufacturer’s instructions. The cells were collected 2 days after transfection by centrifugation (9100×*g*, 1 min, 4 °C), and solubilized by adding 200 µl of buffer containing 50 mM HEPES, pH 7.0, 150 mM NaCl, 1% digitonin (Calbiochem), and protease inhibitor cocktail (Roche) for 1 h at 4 °C. After removing the insoluble material by ultracentrifugation (86,000×*g*, 20 min, 4 °C), 150 µl portions of the supernatant were applied to a Superose 6 Increase 10/300 GL column (GE Healthcare), pre-equilibrated with buffer containing 10 mM HEPES, pH 7.0, 150 mM NaCl, and 0.1% digitonin, and the GFP fluorescence was monitored using an RF20Axs fluorescence detector (Shimadzu). The expression of the EWI-2 mutants was analyzed by western blotting. The whole cell extracts of the transfected cells were fractionated by SDS-PAGE under non-reducing conditions and transferred to a polyvinylidene difluoride membrane. After an incubation with Signal Enhancer HIKARI (nacalai tesque) for 1 h, the membrane was incubated with anti-FLAG M2 monoclonal IgG (SIGMA, F3165) (1:3000) for 30 min and, after washing, further incubated with horseradish peroxidase-conjugated anti-mouse polyclonal IgG (Bio-Rad, STAR207P) (1:3000) for 30 min. Blots were washed with Signal Enhancer HIKARI three times and developed with Chemi-Lumi One (nacalai tesque) according to the manufacturer’s protocols.

### Animal experiments

Neither randomization nor blinding was used for the selection of animals. All animal experiments were approved by the Animal Care and Use Committee of the Research Institute for Microbial Diseases, Osaka University (Osaka, Japan).

### Animals

The *Cd9*-disrupted mice (*Cd9* KO) were a generous gift from Dr. E. Mekada^7^. Hybrid B6D2F1 male and female mice were purchased from Japan SLC. All animals were >8 weeks old when used.

### Reporting summary

Further information on research design is available in the Nature Research Reporting Summary linked to this article.

## Supplementary information

Supplementary Information

Description of Additional Supplementary Files

Reporting Summary

Supplementary Movie 2

Supplementary Movie 1

## Data Availability

Data supporting the findings of this manuscript are available from the corresponding authors upon reasonable request. A reporting summary for this Article is available as a Supplementary Information file. The source data underlying Fig. 5b and Supplementary Fig 7a are provided as a Source Data file. The atomic coordinate for the CD9 crystal has been deposited in the Protein Data Bank, with the accession codes PDB 6K4J. X-ray diffraction images are available at Zenodo data repository (10.5281/zenodo.3716303). Cryo-EM density maps of the complex structures have been deposited in the Electron Microscopy Data Bank, with the accession codes EMD-30026 and EMD-30027 for CD9-EWI-2 and CD9-Fab-EWI-2, respectively.

## Source data

Source Data
