# Supplementary material for: Structural insights into tetraspanin CD9 function
Source: Nat Commun. 2020 Mar 30;11:1606. doi: 10.1038/s41467-020-15459-7 (PMC7105497; doi:10.1038/s41467-020-15459-7)
Supplement: Supplementary file 1 — Supplementary Information [file 41467_2020_15459_MOESM1_ESM.pdf]

Structural insights into tetraspanin CD9 function

Umeda *et al*.

Supplementary Figures 1-8

Supplementary Tables 1-2

Other Supplementary Material for this manuscript includes the following:

Supplementary Movies 1-2

Source Data

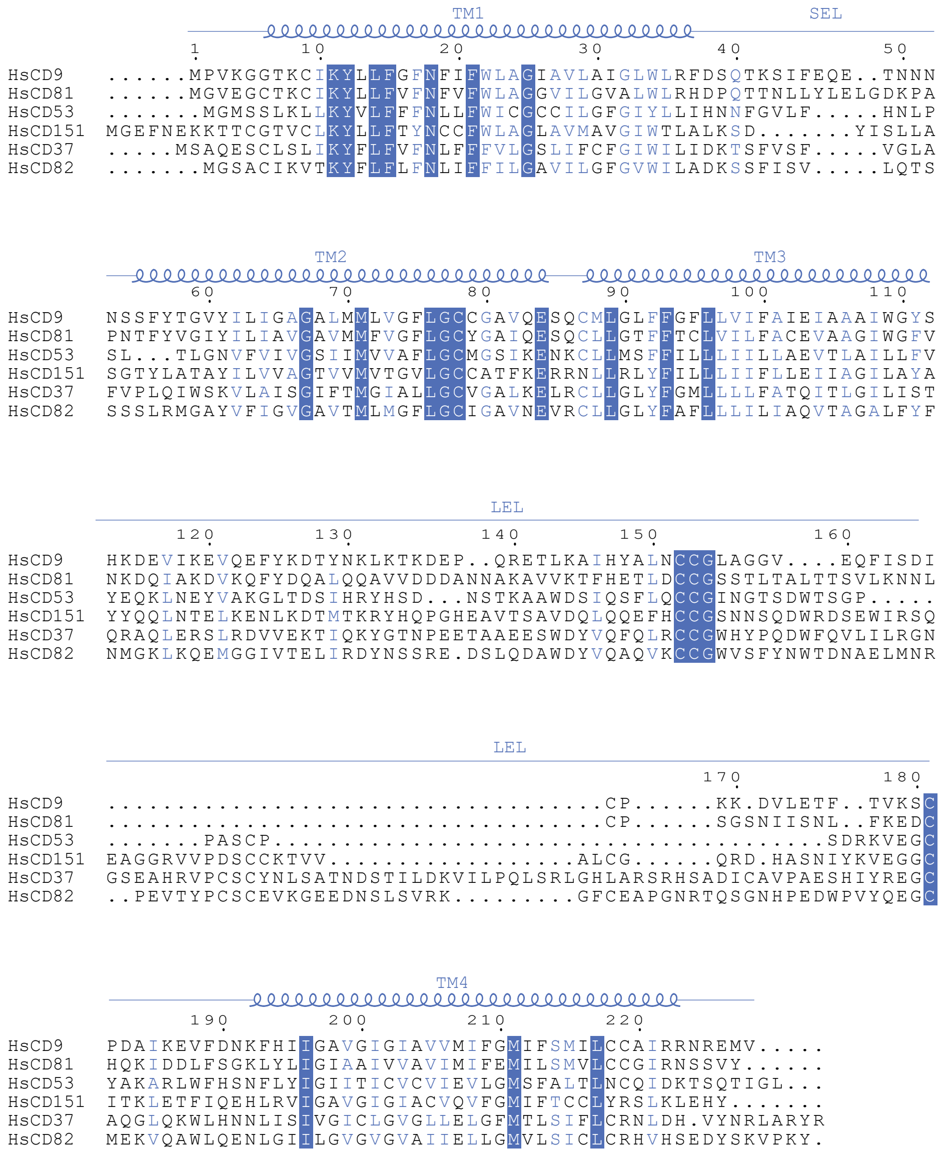

Supplementary Figure 1 │ Sequence alignment of tetraspanins.

The amino acid sequences of human CD9 (UniProt accession code: P21926), CD81 (P60033), CD53 (P19397), CD151 (P48509), CD37 (P11049), and CD82 (P27701) are aligned. Sequence conservation is indicated by blue panels and letters.

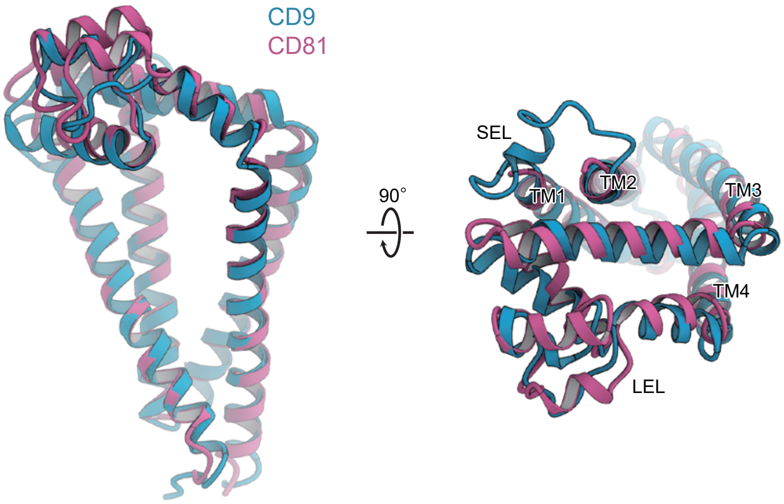

Supplementary Figure 2│Structural comparison with CD81.

Structures of CD9 (cyan) and CD81 (punk) were superimposed together on the transmembrane region. Root mean square deviations for the TM region and the LEL are 0.99 and 2.2 Å, respectively. The SEL was not modeled in the previous CD81 structure.

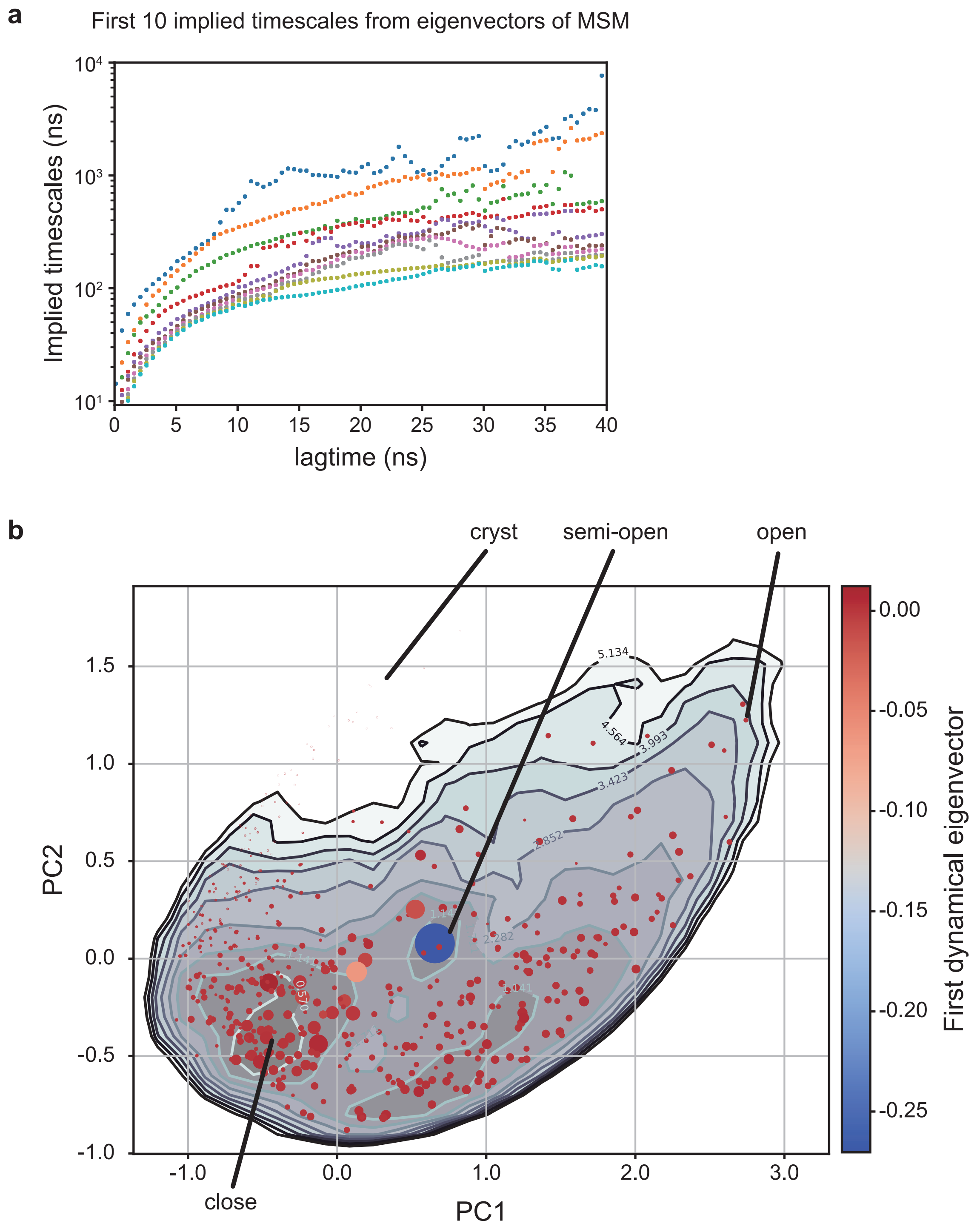

Supplementary Figure 3 | Markov state model analysis.

a, Plot for the lag-time of the Markov state model vs. its first 10 slowest implied timescale. From this plot, we determined a lag-time of 20 ns, as the system exhibits Markovian behavior during this lag-time. **b**, Free energy map shows three distinct states during the simulation. The energy map was shown in the feature space projected onto the 2-dimensional space of principal components (PC) 1 and PC2. Each dot represents the cluster center in the k-means clustering. The color of the dots corresponds to the value of the first dynamical eigenvector, and the size of the dots corresponds to the population size of the cluster. The free energy value was calculated with kernel density estimation (KDE). The four representative conformations are indicated in the energy map.

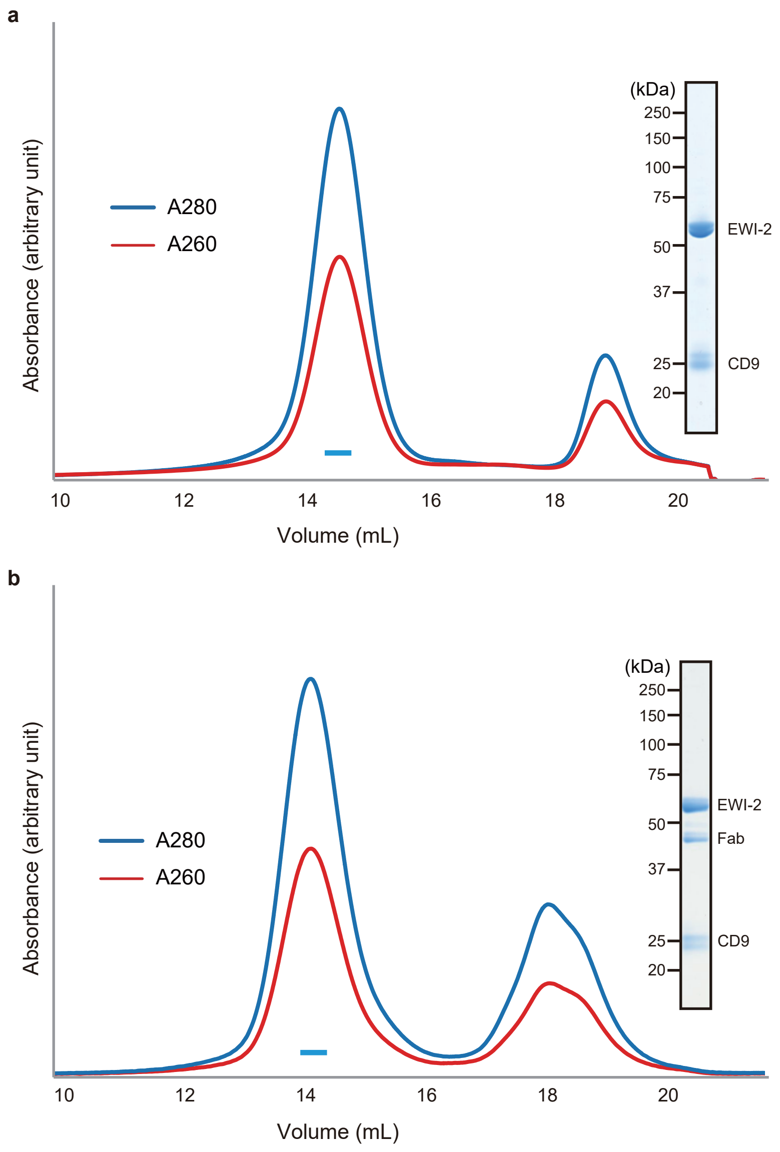

Supplementary Figure 4 | Purification of CD9 in complex with EWI-2.

a, **b**, Representative traces of size-exclusion chromatography for CD9 in complex with EWI-2 (**a**), and the same complex with the anti-CD9 Fab fragment (**b**). The insets show the non-reduced SDS-PAGE analysis of the purified samples. Similar results were obtained at least twice for each complex.

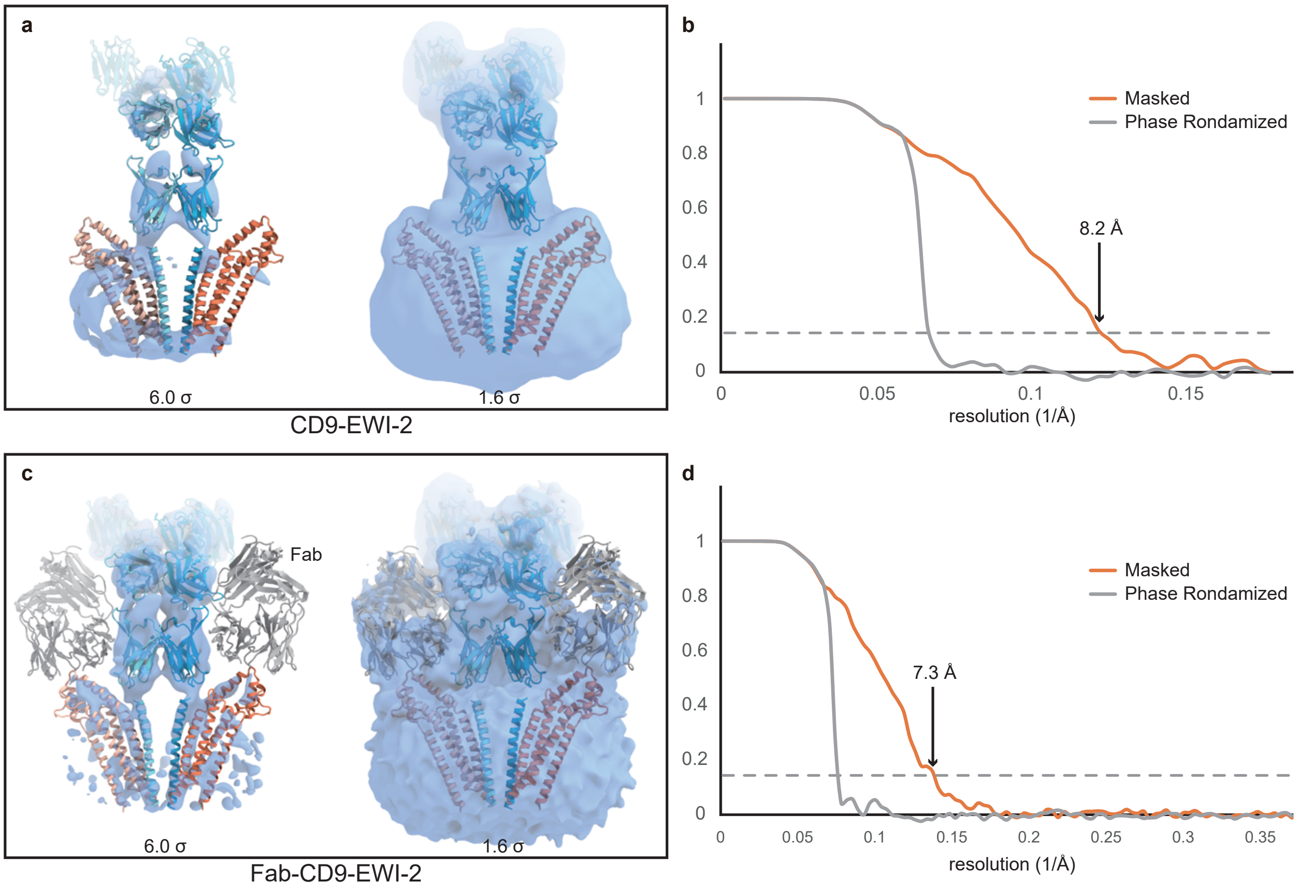

Supplementary Figure 5│Cryo-EM maps of CD9 in complex with EWI-2 and anti-CD9 Fab fragment.
a-**d**, Cryo-EM maps of CD9-EWI-2 (**a**) and Fab-CD9-EWI-2 (**c**), contoured at two different levels. Masked Fourier Shell Correlations (FSC) as a function of the resolutions (**b**, **d**) indicate the resolution of the respective maps. Masked and randomized FSC curves are indicated in orange and grey, respectively. In the CD9-EWI-2 map, the TM helices are not visible, while the extra densities for the LEL region are visible in the low-contoured map. The TM helices of both CD9 and EWI-2 are visible in the Fab-attached complex, even though the Fab densities are very weak and only visible in the low-contoured map.

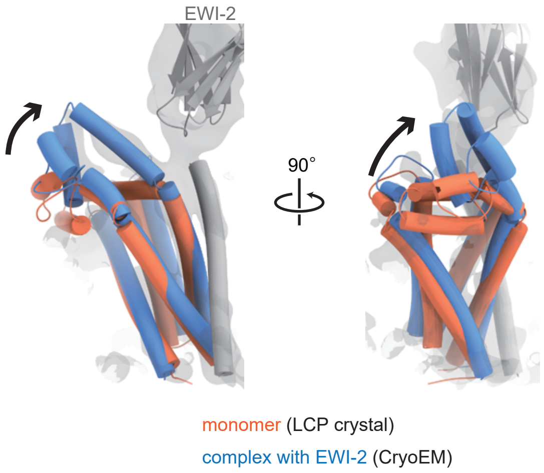

Supplementary Figure 6 | Structural comparison of the monomer and complex structures of CD9.
The monomer CD9 structure of the LCP crystal (red) was superimposed on the cryo-EM structure of the CD9-EWI-2 complex (CD9 and EWI-2 are blue and gray, respectively), revealing the conformational change in the LEL region, while the TM helices superimposed well together.

a

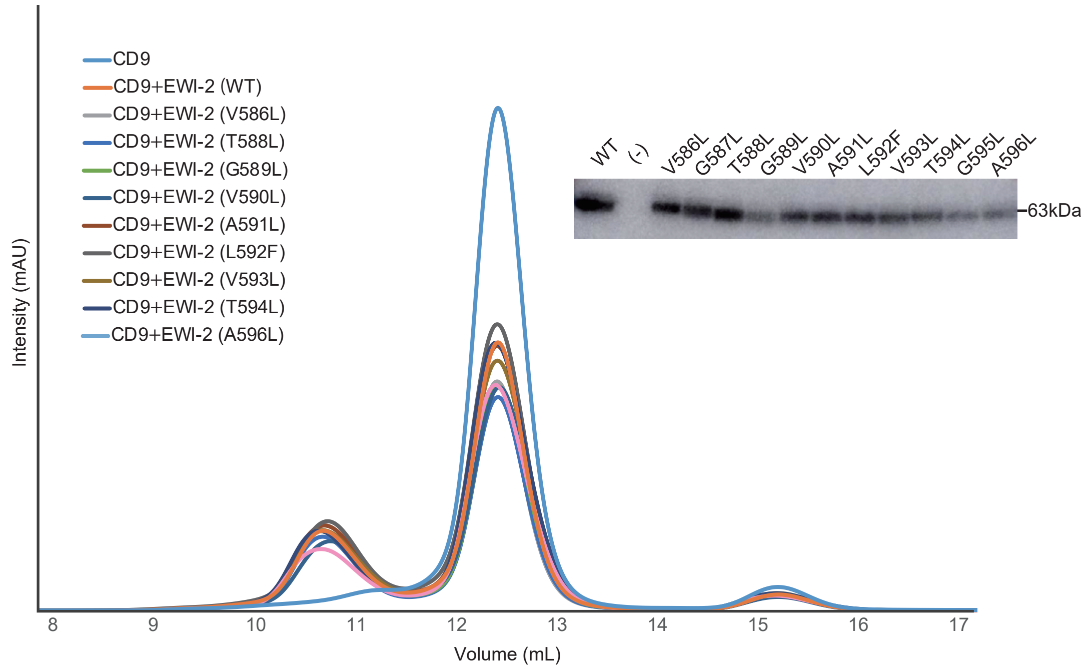

b

```
EWI-2  560  AGSARSGPVTVYPYMHALDTLFVPLLVGTGVALVTGATVLGTITCCFMKRLRKR.................. 613
EWI-F  809  EAEIHSKPVFITVKMDVLNAFKYPLLIGVGLSTVIGLLSCLIGYCSSHWCCKKEVQETRRERRRLMSMEMD 879
```

Supplementary Figure 7│Complex formation of the EWI-2 TM mutants with CD9.

a, Complex formation of the EWI-2 mutants in the membrane-spanning region, analyzed by FSEC. Except for the two glycine residues in Fig. 4D, the other mutations do not affect the complex interaction with CD9. The inset shows the expression levels of the mutants, analyzed by western blotting. The same experiments are repeated at least twice. Three The original uncropped image is also provided as a Source Data file. **b**, Amino acid sequences of the membrane-spanning region of the EWI family proteins that associate with CD9 and CD81. The TM regions are highlighted in yellow backgrounds, and the glycine residues important for the CD9 association are indicated by arrowheads. Cysteine residues that undergo the palmitoyl modification are highlighted in orange panels.

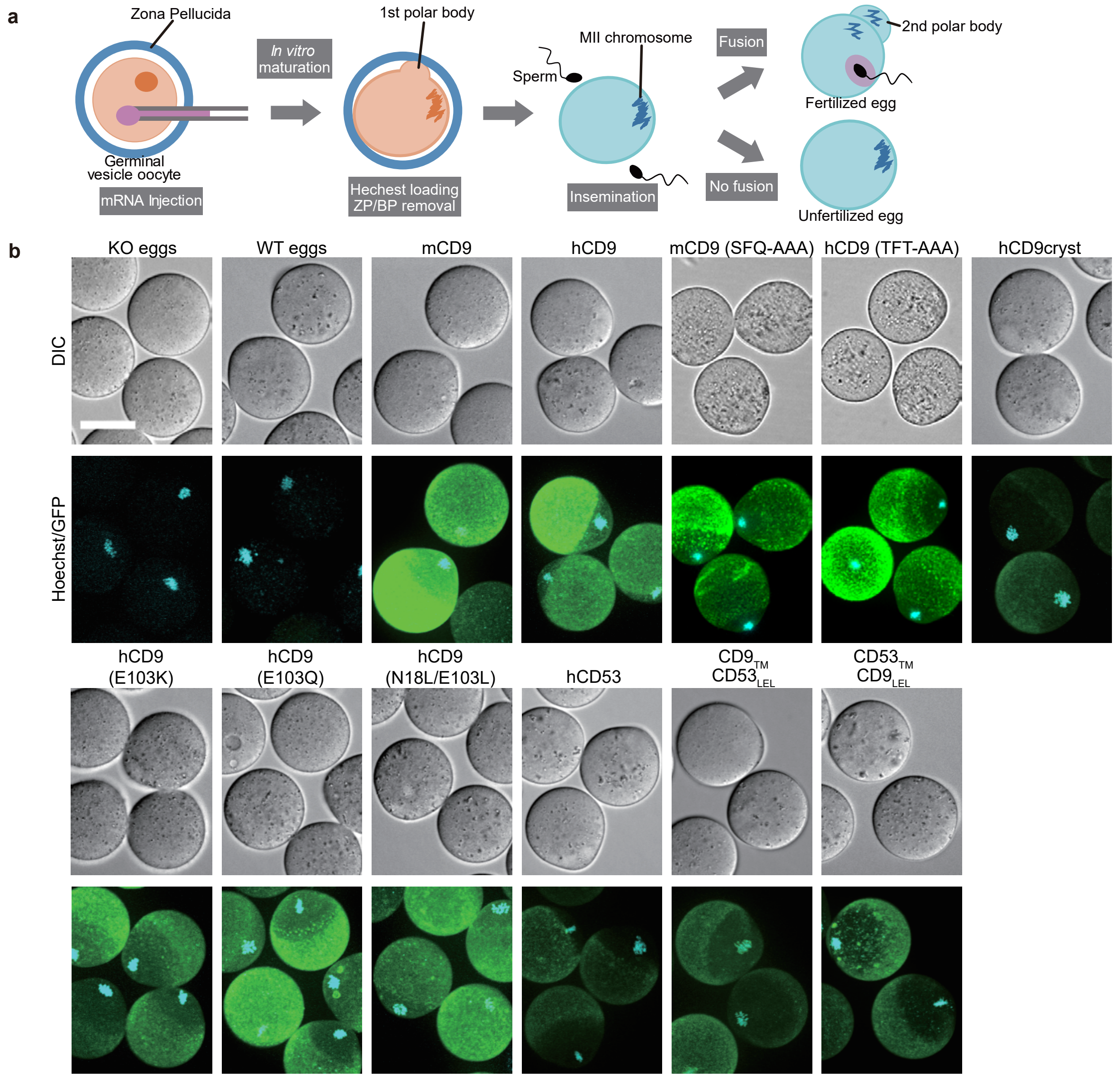

c

Egg genotype	mRNA	Number of experiments	Number of inseminated eggs	Fertilized rate (averaged %)	p-value to KO
WT	None	3	28	86.3	0.0000
KO	None	20	150	10.5	-
KO	*mCD9*	4	44	83.7	0.0000
KO	*hCD9*	7	38	71.0	0.0000
KO	*mCD9 (SFQ-AAA)*	3	36	74.4	0.0000
KO	*hCD9 (TFT-AAA)*	3	44	79.2	0.0000
KO	*hCD9cryst*	6	61	62.6	0.0000
KO	*hCD9 (E103K)*	5	64	61.0	0.0000
KO	*hCD9 (E103Q)*	5	62	66.0	0.0000
KO	*hCD9 (N18L/E103L)*	4	48	45.3	0.0008
KO	*hCD53*	3	35	10.4	0.9905
KO	*CD9-CD53LEL*	3	46	10.6	0.9901
KO	*CD53-CD9LEL*	3	45	34.4	0.0036

Supplementary Figure 8│Sperm–egg fusion complementation assay.

a, Schematic showing the sperm–egg fusion complementation assay. After mRNA injection and in vitro maturation, Hoechst 33342 was loaded to visualize the MII chromosome and the fused sperm heads. **b**, Surface expression of various EGFP tagged CD9 constructs (green), examined by confocal microscopy. Merged images with MII chromosomes stained with Hoechst 33342 (cyan) are indicated. EGFP fluorescence was examined for every oocyte. Similar results were independently obtained at least three times for each mutant. Scale bar, 50 μm. **c**, A table showing numbers of experiments, inseminated eggs, and fertilized eggs. The averaged fertilization rates of repetitive experiments and *p*-values for two-tailed unpaired student's t-test are shown.

Supplemental Table 1 Data collection and refinement statistics of CD9 crystals

	$CD9_{cryst}$ (native)	$CD9_{cryst}^{I20C}$ (Hg-derivatized)
**Data Collection***	BL32XU	BL32XU
Space group	$C222_1$	$C222_1$
Number of crystals	25	47
Oscillation range per crustal (°)	10-180	10-180
Cell dimensions		
a, *b*, *c* (Å)	45.18, 124.83, 129.23	45.18, 125.21, 129.40
α, β, γ (°)	90	90
Wavelength	1.0	1.0
Resolution (Å)	50-2.70 (2.86-2.70)	50-3.17 (3.28-3.17)
R_{meas}	0.518 (3.199)	0.414 (3.152)
R_{pim}	0.067 (0.409)	0.086 (0.657)
$CC_{1/2}$	0.998 (0.515)	0.995 (0.666)
$I / \sigma I$	15.8 (1.6)	11.4 (1.4)
Completeness (%)	100.0 (100.0)	100.0 (100.0)
Redundancy	57.93	22.70
**Refinement**	(PDB 6K4J)	
Resolution (Å)	42.5-2.70 (2.84-2.70)	
No. reflections	10431 (1043)	
R_{work} / R_{free}	0.262/0.289 (0.337/0.423)	
No. atoms		
Protein	1673	
ligand	43	
solvent	13	
***B*-factors**		
Protein	58.6	
ligand	62.7	
solvent	49.8	
**R.m.s. deviations**		
Bond lengths (Å)	0.002	
Bond angles (°)	0.45	
**Ramachandran plot**		
Favored (%)	95.9	
Allowed (%)	4.1	
Disallowed (%)	0	

* Statistics for data collection is partly derived from our previous report[11].

** Values in parentheses are for highest-resolution shell.

Supplemental Table 2 Data collection statistics for cryo-EM single particle analysis

	CD9-EWI-2 (EMD-30026)	CD9-EWI-2-Fab (EMD-30027)
**Data collection and processing**		
Microscope	Talos Arctica (FEI)	
Voltage (kV)	200	
Detector	K2 Summit (Gatan)	
Data acquisition software	EPU	
Magnification	×10,000	×10,000
Pixel size (Å)	1.351	1.351
Electron exposure (e^{-} $Å^{-2}$)	50	50
Defocus range (μm)	1.0-2.0	1.0-2.0
Micrographs collected (no.)	1,398	2,360
Initial particle images (no.)	276,879	542,954
Final particle images (no.)	199,267	124,701
Symmetry imposed	C1	C1
Map resolution (Å)	8.2	7.3
FSC threshold	0.143	0.143
