# Supplementary material for: Structural insights into tetraspanin CD9 function
Source: Nat Commun. 2020 Mar 30;11:1606. doi: 10.1038/s41467-020-15459-7 (PMC7105497; doi:10.1038/s41467-020-15459-7)
Supplement: Supplementary file 2 — Description of Additional Supplementary Files [file 41467_2020_15459_MOESM2_ESM.pdf]

Title: Supplementary Movie 1

Description: Ribbon representation of the CD9 MD simulation.

A movie of the MD simulation corresponding to 1 μs timescale is shown with ribbons.

Title: Supplementary Movie 2

Description: Ribbon representation of the CD9 MD simulation.

A movie of the MD simulation corresponding to 1 μs timescale is shown with surface representation. The molecular surface is drawn with the Quicksurf feature in VMD[43]
